# Modification of the Creator recombination system for proteomics applications – improved expression by addition of splice sites

**DOI:** 10.1186/1472-6750-6-13

**Published:** 2006-03-06

**Authors:** Karen Colwill, Clark D Wells, Kelly Elder, Marilyn Goudreault, Kadija Hersi, Sarang Kulkarni, W Rod Hardy, Tony Pawson, Gregg B Morin

**Affiliations:** 1 Samuel Lunenfeld Research Institute, Mount Sinai Hospital, Toronto, Ontario, Canada; 2 Department of Medical Genetics and Microbiology, University of Toronto, Toronto, Ontario, Canada; 3 Michael Smith Genome Sciences Centre, BC Cancer Agency, Vancouver, BC, Canada

## Abstract

**Background:**

Recombinational systems have been developed to rapidly shuttle Open Reading Frames (ORFs) into multiple expression vectors in order to analyze the large number of cDNAs available in the post-genomic era. In the Creator system, an ORF introduced into a donor vector can be transferred with Cre recombinase to a library of acceptor vectors optimized for different applications. Usability of the Creator system is impacted by the ability to easily manipulate DNA, the number of acceptor vectors for downstream applications, and the level of protein expression from Creator vectors.

**Results:**

To date, we have developed over 20 novel acceptor vectors that employ a variety of promoters and epitope tags commonly employed for proteomics applications and gene function analysis. We also made several enhancements to the donor vectors including addition of different multiple cloning sites to allow shuttling from pre-existing vectors and introduction of the lacZ alpha reporter gene to allow for selection. Importantly, in order to ameliorate any effects on protein expression of the loxP site between a 5' tag and ORF, we introduced a splicing event into our expression vectors. The message produced from the resulting 'Creator Splice' vector undergoes splicing in mammalian systems to remove the loxP site. Upon analysis of our Creator Splice constructs, we discovered that protein expression levels were also significantly increased.

**Conclusion:**

The development of new donor and acceptor vectors has increased versatility during the cloning process and made this system compatible with a wider variety of downstream applications. The modifications introduced in our Creator Splice system were designed to remove extraneous sequences due to recombination but also aided in downstream analysis by increasing protein expression levels. As a result, we can now employ epitope tags that are detected less efficiently and reduce our assay scale to allow for higher throughput. The Creator Splice system appears to be an extremely useful tool for proteomics.

## Background

After the publication of the human genome in 2001 ([1,2]), the focus shifted from gene identification to understanding the function of the identified gene products. Although the human genome project predicted and annotated genes, cDNAs for experimental use were still only available in small numbers. Thus, a number of large scale human cDNA cloning projects were established including MGC ([3])[4], Kazusa ([5])[6], Nedo [7,8], and the German Human cDNA project [9]. The availability of these cDNA collections has facilitated rapid progress in the study of the human proteome. These cDNAs, however, contain 5' and 3' untranslated regions (UTRs) that preclude using them directly to make fusion proteins for downstream applications. Thus, several groups are creating human open reading frame (ORF) libraries, including the Harvard Institute of Proteomics and the Vidal lab at Harvard (reviewed in [10,11]). These large scale ORF cloning efforts and other smaller projects employ *in vitro *recombination cloning to allow rapid DNA shuttling between a storage vector and various expression vectors that add 5' or 3' sequences that encode epitope tags or proteins which enable investigation of protein function. Elledge developed the first recombination system using the Cre recombinase ([12]), the non-commercial Univector system. The two most widely used commercial recombinational systems are the Gateway system by Invitrogen and the Creator system from Clontech, which are generally used by the large scale cDNA cloning programs.

The Creator system requires only the Cre recombinase from Bacteriophage P1 and loxP, its recognition sequence, for the recombination reaction ([13], reviewed in [10]). Initially, ORFs are cloned between two loxP sites in a donor vector. Then, sequences flanked by these two loxP sites are recombined by Cre recombinase into a single loxP site on an acceptor vector. However, the recombination site is regenerated during the reaction leading to undesirable downstream products. To circumvent this problem, the correct clone is selected by employing a positive/negative selection scheme. Any recombinant vector that still retains the donor backbone is negatively selected due to the presence of the *sacB *gene from *Bacillus subtilis *that is lethal to *E. coli *when its substrate, sucrose, is added to the media [14,15]. The desired recombinant vector is positively selected by reconstitution of the Chloramphenicol resistance (CmR) gene when the CmR ORF on the donor vector properly aligns with the CmR promoter on the acceptor vector. In mammalian cells, a splicing event engineered into the Creator system removes the CmR gene to allow for 3' tagging.

Our laboratory chose the Creator system as our primary recombinational system because of its ready adaptability, minimal cost, and performance. We subsequently enhanced this system to better suit the requirements of our laboratory. First, to allow for easy cloning of ORFs into donor vectors, we modified the multiple cloning site on the donor vector to possess two restriction endonuclease sites that occur infrequently in mammalian cDNAs. Second, various acceptor vectors were created to facilitate different expression technologies for proteomics applications. Lastly, mammalian pre-mRNA splicing sequences were introduced into the vectors so that linker sequences between 5' tags and the ORFs would be removed upon pre-mRNA processing. We term this new system 'Creator Splice'. Interestingly, the addition of an intron at the 5' end increased overall expression, probably due to better processing of the mRNA. These new adapted vectors are a good resource for common proteomic applications.

## Results

### Optimization of the Creator recombination reaction

To facilitate high-throughput ORF manipulation, a cost-effective and robust method of DNA transfer is paramount. The recombination between loxP sites in the Creator system is executed by only one protein (Cre). Since Cre enzyme can be readily produced in a typical laboratory, employment of the Creator system can result in significant cost savings. From a 600 ml starting bacterial culture expressing His-tagged Cre, enough enzyme was purified to perform over 20,000 reactions. While testing the His-Cre enzyme, we discovered that altering the recombination buffer composition had a marked effect on the number of colonies recovered after transformation of the Cre reaction into bacteria – a step that is traditionally inefficient. By using the optimized buffer (see methods), the number of colonies after transformation increased greater than 5 fold over the standard buffer (data not shown).

### Modification of donor vectors

**Figure 1:**
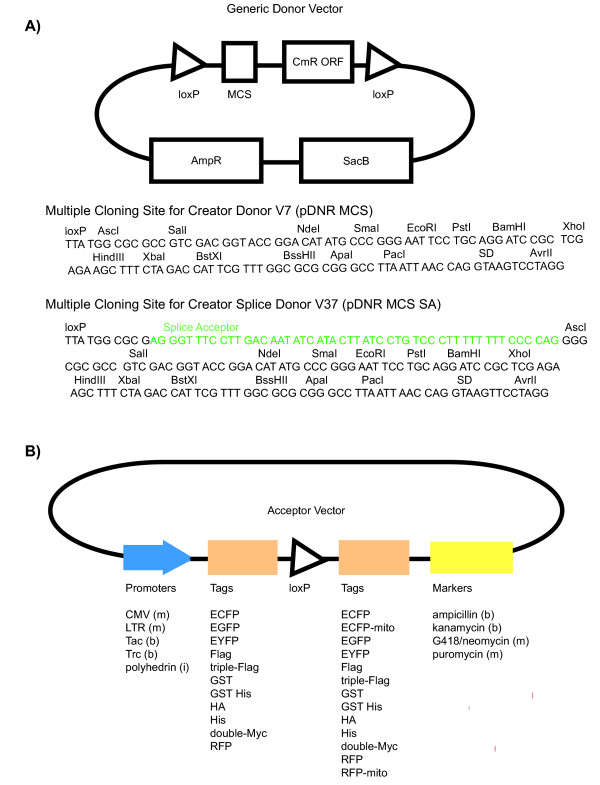
Description of vectors for the Creator and Creator Splice systems. A) Schematic of the donor vector illustrating the essential features of the donor vector. ORFs are cloned into the Multiple Cloning Site (MCS) that is flanked by two loxP sites. The CmR ORF following the MCS, but still within the two loxP sites, ensures that both the ORF cassette from the donor vector and the acceptor vector backbone (containing the CmR promoter) are retained in the final product. The ampicillin resistance gene (AmpR) provides positive selection and the SacB gene imparts negative selection (against the donor backbone). Sequences of the novel MCS for the modified Creator and Creator Splice vectors are indicated below the diagram. The MCS starts at the AscI site and ends at the PacI site. The last four nucleotides of the loxP site are shown for orientation. The splice acceptor sequence in the Creator Splice vector is shown in green to emphasize the difference between the two vectors. B) A schematic of the modular nature of the acceptor vectors. The types of cassettes (promoter, tags, selectable markers) that can be interchanged to create vectors for specific proteomics needs are illustrated, not all combinations are available (Table 2). Abbreviations: AmpR – ampicillin resistance gene, b – bacteria expression, CmR – chloramphenicol resistance ORF, i – insect expression, m – mammalian expression, MCS – multiple cloning site, SA – splice acceptor.

To reduce the cost and increase the efficiency of moving ORFs into the Creator donor vectors, several changes were made to the donors. The multiple cloning site (MCS) of donor pDNR Dual vector (Clontech) does not contain any rare-cutting restriction enzyme sites. Accordingly, we were unable to standardize cloning protocols using the traditional ligation route since the restriction enzyme sites in the MCS of the donor vector are often present in ORF sequences. Thus, the MCS of pDNR Dual was altered to add an AscI site to the 5' end and a PacI site to the 3' end (Figure 1A). These two sites were chosen for several reasons. First, the 8-base recognition sites for both enzymes occur infrequently (95 % of 25,000 mouse cDNAs examined did not contain recognition sites for either of these enzymes). Second, AscI and PacI are compatible in the same restriction enzyme buffer and do not exhibit star activity. Third, both enzymes are active against various forms of DNA including linear, supercoiled and bacterially methylated DNA (neither site contains a methylation sequence). Fourth, both enzymes digest DNA at a non-degenerate and non-interrupted recognition site and cleavage results in a 5' or 3' DNA overhang. Fifth, both enzymes efficiently cleave DNA from a wide variety and quality of preparations. Sixth, AscI has a strong consensus to the Kozak sequence needed for mammalian expression (GCGCC vs. CCACC) [16,17]. Therefore, it was not necessary to add additional sequences to each construct to conform to the Kozak consensus. Lastly, the PacI site contains stop codons for the two non-reading frames. The resulting vector, that also removes a hexa-histadine tag at the C-terminus, is called pDNR MCS (V7) (Table 1). In addition, we have constructed other donor vectors with different multiple cloning sites: pDNR MCS BE (V624) with BamHI, SmaI, and EcoRI sites compatible with pGEX series vectors (Amersham) and pDNR MCS ES (V795) with EcoRI, NotI and SalI sites (Table 1). We also inserted the lacZ alpha sequence into the MCS of our donor vectors (V308, V677). The lacZ alpha sequence reduces the time to screen for positive clones as blue colonies indicate undigested wildtype vector.

### Acceptor vector construction

**Figure 2:**
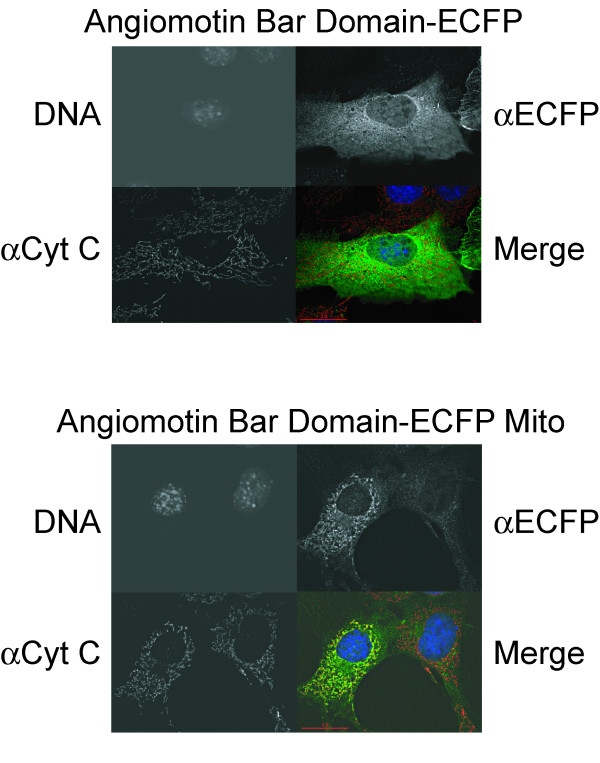
The Mito tracking peptide targets proteins to the mitochondria. NIH 3T3 cells were transiently transfected with vectors expressing either the N-terminus of Angiomotin fused to a C-terminal ECFP (top panel) or the same protein fused to a C-terminal ECFP plus a mitochondrial tracking peptide (ECFP-mito) (bottom panel). The cells were fixed and stained with anti-ECFP (green colour in merge) that recognizes the fusion construct or anti-Cytochrome C (red colour in merge) to mark the mitochondrial membrane. DNA is stained with Hoescht (blue colour in merge). The bottom right image on both panels is a composite of all three stainings. Note that only transfected cells will display ECFP staining. Abbreviations – mito – Mito tracker, Cyt C – Cytochrome C, ECFP – enhanced cyano fluorescent protein.

The utility of recombinational cloning systems lies in the availability of acceptor vectors for the tasks at hand. To this end, we have created over 20 acceptor vectors with different tags, promoters, and resistance markers (Figure 1B). For expression of proteins in mammalian cells that can be easily purified by immunoprecipitation, visualized in lysates by immunoblot analysis or in cells by immunofluorescence, we have created vectors containing the following epitope tags: Single and Triple-Flag, Double-Myc, and HA (Table 2). In addition, we inserted a cassette containing a 5' Triple-Flag tag and loxP site into the pMSCV retrovirus vector. For expression in non-mammalian cells, we created acceptor vectors for both bacterial (GST or His tags) and baculoviral expression (GST and His tag). For localization using proteins fused to fluorescent proteins, we use vectors pLP ECFP C1 (enhanced cyano), pLP EGFP C1 (enhanced green), pLP EYFP C1 (enhanced yellow) and pLPS-3'EGFP and we also created a 3' ECFP vector. To expand our repertoire of fluorescent proteins, we mutated the red fluorescent protein gene from Clontech (dsRED2) to increase the fluorescent intensity and prevent homodimerization of the resulting red fluorescent protein (RFP) [18,19]. Acceptor vectors containing both 5' and 3' RFP have subsequently been created. We also constructed vectors that fuse a fluorescent protein and the mitochondrial targeting sequence of *Listeria monocytogenes *ActA protein (Mito tracker) ([20]) to the COOH terminus of the expressed insert. Proteins fused at their COOH terminus to the Mito tracker are re-located to the mitochondria ([21,22]). In Figure 2, the first 240 amino acids of the protein Angiomotin fused at the C-terminus to ECFP displayed a cytoplasmic expression (top panel). However, when the same protein sequence was fused to the ECFP-mito tracker, it was redirected to the mitochondria and co-localized with Cytochrome C (bottom panel). This vector can be used to verify protein-protein interactions *in vivo *by demonstrating that the re-localization of one protein leads to the re-localization of its partner(s). For example, the SH3 domain of TUBA when fused to the mito tracker will recruit actin to the mitochondria [22]. In addition, recruitment of the partner(s) away from their endogenous location may lead to phenotypic consequences for the cell that can aid functional interpretation.

### Addition of splice sites to the Creator system (Creator Splice)

**Figure 3:**
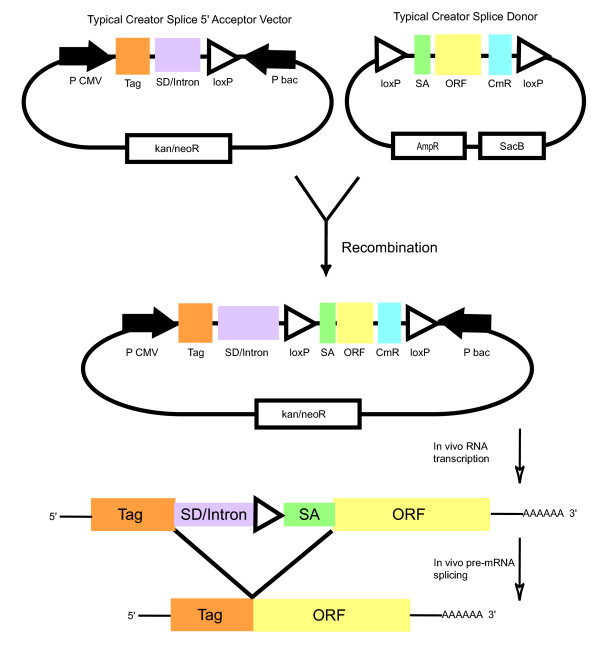
Schematic of a typical recombination reaction in the Creator Splice system. The SD/intron, shown in purple, is the only difference between a Creator Splice acceptor and a standard acceptor vector. A Creator Splice donor and acceptor are recombined in the presence of Cre recombinase *in vitro*. An intron is formed in the resulting expression vector starting from the SD/intron supplied by the acceptor and ending with the splice acceptor from the donor vector. Upon transfection and transcription in mammalian cells, the intron is removed and the tag is juxtaposed onto the ORF. Abbreviations: bac – bacteria promoter, CmR – Chloramphenicol resistance ORF, kan/neoR – kanamycin and neomycin resistance gene, P – promoter, SA – splice acceptor, SD – splice donor.

One issue with recombinational systems is the large linker between the tag and the ORF after recombination due to the recombination site and other intervening sequences. For 5' tags in the Creator system, the linker size can be between 51 to 81 nucleotides (17–27 codons) depending on which two vectors are recombined. In contrast, the splicing event employed for 3' tags to remove the CmR gene can introduce as little as 21 nucleotides (7 codons). To reduce the linker size for 5' tags, we added splicing signals similar to that utilized for 3' tags. In order to have a functional intron after recombination, the 3' splice acceptor sequence must come from the donor vector and the 5' splice donor and intron sequences must come from the acceptor vector (Figure 1A and 3). For the 5' intron, we selected 360 nucleotides from the Adenovirus L1 major late intron before the 2^nd ^leader. This intron does not undergo alternative splicing, does not contain any potential splice donors or splice acceptors, and is unlikely to contain sequences that would reduce splicing efficiency (analyzed by BDGP splice site prediction [23,24]). We purposely chose a small intron size because smaller introns tend to be more abundant in higher expressed mRNAs [25]. For the donor vectors, we added splice acceptor sequences preceding the MCS (V37, V309, V678, V694, Figure 1A, Table 1). We also chose the splice acceptor from the same Adenovirus major late intron used above. Both the splice donor and splice acceptor sequences have maximal scores for splice site consensus sequences as defined by the BDGP splice site prediction algorithm. Upon recombination and expression in mammalian cells, the resulting pre-mRNA will undergo splicing (Figure 3). The amount of sequence between the tag and the resulting protein is now reduced to between 3 and 5 amino acids in the Creator Splice system depending on the vectors recombined. Three potential splice acceptor sites were also identified using the BDGP splice site algorithm in the CmR ORF. Since these cryptic sites might reduce protein levels when using a C-terminal tag, we constructed a new vector that introduced silent mutations in the CmR ORF to remove the two strongest potential sites (V954). However, removal of these sites did not increase protein levels of 3' tags as analyzed by immunoblot analysis (data not shown).

### Expression of constructs in mammalian cells

**Figure 4:**
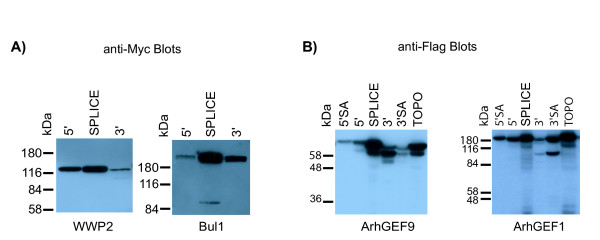
Protein expression levels in the Creator and Creator Splice system. The indicated constructs were transiently transfected into HEK293T cells. After 48 hours, the cells were lysed and the lysate subjected to immunoblot analysis. A) WWP2 and BulI fused to Double-Myc epitope tags were detected by Myc antibody 9E10. B) ArhGEF9 and ArhGEF1 fused to Triple-Flag tags were detected by Flag antibody M2. Abbreviations: 5' SA – Vector created by recombining a Creator Splice donor vector, (splice acceptor at 5' end – V37-based), with a 5' tag acceptor vector lacking a splice donor/intron (V179). No splicing occurs in these transcripts, 5' – Vector created by recombining a donor lacking a 5' splice acceptor (V7 or V308-based) and a 5' tag acceptor vector lacking a splice donor/intron (V516- Double Myc, V179 – Triple Flag). No splicing occurs in these transcripts, SPLICE – Expression vector created by recombining a Creator Splice donor (5' splice acceptor – V37-based) and a Creator Splice 5' tag acceptor vector containing a splice donor/intron (V517 – Double Myc, V180 – Triple Flag). Resulting transcripts undergo splicing at the 5' end to remove the loxP sequence and juxtapose the tag onto the ORF, 3' – Vector created by recombining a donor lacking a 5' splice acceptor (V7 or V308-based) with a 3' tag acceptor vector lacking a splice donor/intron (V518 – Double Myc, V181 – Triple Flag). Splicing only occurs at the 3' end to remove the CmR gene (The donor contains a splice donor preceding the CmR ORF and the acceptor vector contains a splice acceptor preceding the tag), 3' SA – Vector created by recombining a Creator Splice donor vector containing a 5' splice acceptor (V37-based) and a 3' tag acceptor vector lacking a splice donor/intron (V181 -Triple Flag). Splicing only occurs at the 3' end to remove the CmR gene (same as 3' tag) and TOPO – 5' tag, non-Creator system.

While the ease of rapidly shuttling a particular ORF into various vectors is a major merit of *in vitro *recombination systems, protein expression levels from the resulting expression vectors must be sufficiently high for the desired assays. To analyze the expression levels of various acceptor vectors, immunoblot analysis was performed on cell lysates. In Figure 4A, two different proteins (WWP2 and BulI) fused to Double-Myc tags were expressed in HEK293T cells by transient transfection. In this assay, we tested three different tag positions: SPLICE (splicing at 5' end), 5' (no splicing) and 3' (splicing at 3' end to remove the CmR gene). Full-length proteins at the expected sizes were recognized by the Myc antibody 9E10 in all three combinations of tags for both ORFs. Interestingly, the Creator Splice system appeared to have the highest protein expression levels.

To further test our system and to ensure that Creator and Creator Splice systems are compatible, we expressed proteins fused to Triple-Flag epitopes from vectors derived from various recombinations. In addition to examining the three different tag positions analyzed above, we added two more combinations. First, we wanted to confirm that the donor vectors from the Creator Splice system could express proteins when recombined with acceptor vectors from the regular Clontech Creator system (Figure 4B, labeled 5' SA). In these recombinants, the splice acceptor sequence in the donor vector is not removed leading to an extra 18 amino acids between the tag and the ORF. Second, we wanted to ensure that the Creator Splice donor vectors did not interfere with protein expression when using tags at the 3' end (Figure 4B, labeled 3' SA). To these ends, immunoblot analysis was performed on HEK293T cell lysates after transfection with the indicated constructs (Figure 4B). All combinations of tags resulted in full-length protein expression. Therefore, vectors from the Clontech Creator and Creator Splice Systems can be interchanged if necessary. Of note, for the vectors tested in this assay, expression levels of proteins ArhGEF9 and ArhGEF1 seemed highest from the Creator Splice vectors (labeled SPLICE, Figure 4B).

**Figure 5:**
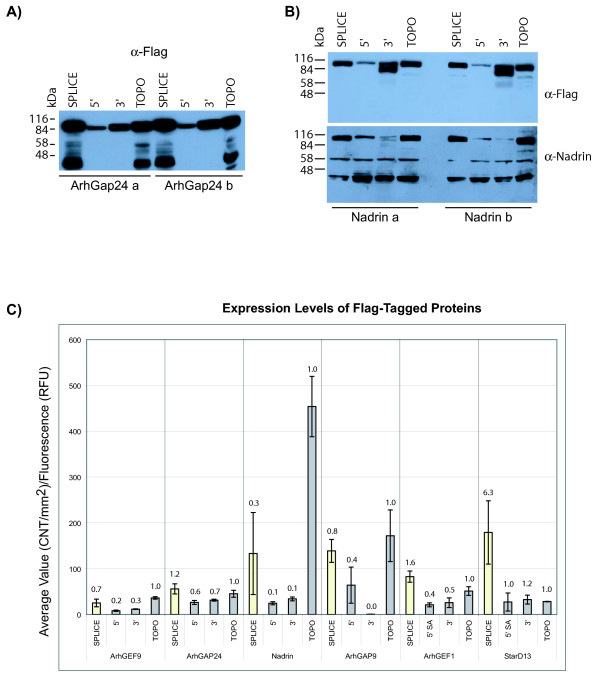
A) Anti-Flag immunoblot of ArhGap24 with tags in the indicated positions. The transfections and expression analyses were performed in duplicates (a and b). B) Immunoblot analysis of Nadrin with tags in the indicated position. The top panel was probed with anti-Flag antibody. The immunoblot was then stripped of antibody and reprobed with anti-Nadrin (bottom panel). C) Quantitative analysis of protein levels for six different proteins with Triple-Flag epitope tags at the indicated positions. For each replicate, the band intensity, as detected by Flag M2 antibody, was normalized by the transfection efficiency as detected by fluorescent values of a co-transfecting plasmid pLP ECFP C1. The normalized band intensity was then average between the two replicates. Ratios of each tagged-protein relative to the protein expressed from the same ORF from the TOPO vector are shown above the bars. Abbreviations: SPLICE – Creator Splice Donor (V37-based) and Creator Splice 5' acceptor (V180) – splicing occurs at 5' end, 5' – standard Clontech system (donor – V7-based and acceptor – V179) – no splicing at either end, 3' – standard Clontech system (donor – V7-based and acceptor – V181) – splicing at 3' end to remove the CmR gene, 5' SA, Creator Splice Donor (V37-based) and 5' acceptor vector (V179) – no splicing at either end, TOPO – 5' tag non-recombination system, CNT – counts, RFU – relative fluorescent units.

To confirm that the Creator Splice system displays superior expression levels, we tested 6 different ORFs for differences in protein expression between the Clontech Creator system, the Creator Splice system, and the Topoisomerase system (Invitrogen, [26]) all using a Triple-Flag epitope as the tag. HEK293T cells were transiently transfected with the indicated constructs and immunoblot analysis with the Flag M2 antibody was performed on the lysates. Representative gels of two proteins (ArhGap24 and Nadrin) are displayed in Figure 5A and 5B. Similar to the results using Double-Myc epitope tags, the Creator Splice system appeared to have the highest levels of expression of the Creator constructs and was comparable to expression from the non-recombinational Topoisomerase vector. One possibility that could account for the differences in expression seen between the 5' Creator tags is that the loxP site is masking to some extent the ability of the epitope to be recognized by the Flag antibody. To test this possibility, we re-probed the anti-Flag immunoblot of Nadrin with an antibody specific to Nadrin (Figure 5B, bottom panel). The Nadrin antibody detects more protein from the Creator Splice construct (SPLICE) than from the 5' Clontech Creator constructs indicating that differences in epitope detection cannot account for the differences in protein expression.

To quantitate protein expression levels, the band intensity of the tagged proteins, as detected by anti-M2 Flag antibody, was analyzed. Only the band corresponding to the size of the full-length protein is quantitated. To control for transfection efficiency, the vector pLP-ECFP C1 that expresses ECFP was co-transfected with the test expression vectors, and the amount of fluorescence in the cell lysates was measured. This control will normalize for differences in transfection levels as well as differences in cell count and lysate volume. A bar chart of the expression levels of various constructs normalized by fluorescence levels is shown in Figure 5C. Only constructs expressed in the Creator Splice system were comparable with constructs cloned via the non-recombination Topoisomerase system. Thus, it appears that the additional sequences introduced by the loxP site in the original Clontech Creator system vectors may reduce expression levels.

## Discussion

We describe here several improvements on the Creator recombinational cloning system that will facilitate the use of the increasingly large number of cDNAs available for proteomics applications. In the Creator System, a DNA fragment can be introduced into the donor vector by standard restriction enzyme digestion and ligation or by the In Fusion recombination method (Clontech). The latter method is used by the large scale cloning laboratory at the Harvard Institute of Proteomics (HIP) for the creation of their FLEX Gene libraries ([10]). While we have used this method to clone into donor vectors with similar success as HIP, it is our system of choice only for inserts larger than 3 kilobases or those with internal AscI or PacI sites. The In Fusion method requires longer oligonucleotides than traditional ligation reactions, more expensive reagents (In Fusion versus restriction enzymes), and is prone to high background if the digested vector preparation is contaminated or if digestion is incomplete. For this reason, we altered the donor vector to incorporate AscI and PacI sites – rare base cutters that would allow us to use the ligation system of cloning in the majority of cases and In Fusion recombination for the exceptions.

The interest in any recombination system will increase as more downstream applications become compatible with the system. To that end, we have made 22 acceptor vectors and are continually increasing our repository. While we have made vectors for expression in bacteria and insect cells, our primary focus is on mammalian expression and protein function analysis. As seen in Figure 1B, the acceptor vectors are modular such that sequences for epitope tags, selectable markers, promoters, and expression systems can be interchanged. One example of an acceptor vector is based on a concept employed by the Gertler lab where a protein of interest is fused both to a fluorescent protein and the mitochondria tracking peptide from *L. monocytogenes *ActA protein. Proteins fused to this peptide are targeted to the mitochondria (Figure 2, [20,22]). Of interest, proteins that bind to the re-targeted protein can also be relocated demonstrating an *in vivo *interaction and possibly leading to phenotypic consequences that can aid in the analysis of protein function [22].

One major advantage of the Creator Splice system described here over the Creator and Gateway recombinational systems is the reduction in linker sequences between the tag and protein. Although we deleted extraneous sequences in the donor and acceptor vectors whenever possible, the major reduction in linker size was via splicing. The Creator Splice system reduces the amount of linker to 3 to 5 amino acids, similar to that introduced between tags and proteins in a typical restriction enzyme/ligation protocol. Since the linker sequence is so small, it is possible to add additional sequences to the acceptor vectors to create the most appropriate linker for downstream applications.

When we compared the Creator Splice system against the standard Clontech Creator system, the Creator Splice system had the highest levels of protein expression. A likely explanation for these increased expression levels is the presence of the 5' intron. There is accumulating evidence that pre-mRNAs containing introns are expressed at higher levels than pre-mRNAs that lack introns [27-30]. Processes involved in mRNA maturation are highly interdependent and the presence of introns influences transcription, polyadenylation, nuclear export and translation [31]. Early steps in the mRNA maturation pathway affect later processing steps by imprinting information on the transcript via protein binding (reviewed in [31,32]. The exon junction complex (EJC), a splicing-dependent complex, is deposited upstream of exon-exon junctions [33,34]. Quantitative analysis of intron effects has demonstrated that more protein is produced per transcript when the transcript has undergone splicing and this increase is dependent on the EJC [28,35-37]. Therefore, the increased protein expression seen with the Creator Splice system is likely due to more efficient processing of the mRNA.

Interestingly, Creator clones with 3' tags also utilize splicing to remove the CmR gene. However, we see reduced expression of these 3' tags as compared to 5' tags in the Creator Splice system. This reduction might result from differences in sequence and position of the introns used for the 5' and 3' tags. Even in identical positions, two distinct introns can have markedly different effects on gene expression [38]. The intron chosen for the Creator Splice system is a bona fide intron from Adenovirus that has likely evolved to be very effectively spliced, in contrast to the longer engineered intron for the 3' tag that contains the CmR gene. We also identified three potential splice acceptors in the CmR ORF that may lead to undesirable splice products, although removal of two of these sites did not alter subsequent protein expression levels. Lastly, the position of the intron can also highly impact protein expression levels and introns proximal to the 5' end enhance transcriptional activity [39,28,41].

Proteins produced from Creator vectors with 5' tags (unspliced) express at lower levels than those produced from a topoisomerase vector with the same 5' tag. This result is mirrored in bacteria where the loxP site is deleterious to protein expression (for example, see Additional File 5F &5G). This decrease may be due to the extra 34 nucleotides of the loxP site in the linker. Alternately, the hairpin secondary structure of loxP (predicted by mFold, [42,43]) may cause the transcription or ribosomal machinery to stall in bacteria [44]. In mammalian cells, the effect that the hairpin would have on mRNA from 5' tagged constructs is not obvious since it is downstream of the initiation codon and would be unlikely to inhibit the ribosomal complex once bound to RNA [45]. Interestingly, the presence of a loxP hairpin upstream of the start codon in mRNA from 3' tagged constructs could adversely affect translation by interfering with 40S ribosomal subunit binding [45].

While many assays do not require or are compromised by high expression levels, it is often easier to reduce expression levels than raise them. This reduction can be accomplished by using a weaker promoter or by transfecting less DNA. However, it is often not feasible to transfect sufficient DNA required to achieve high expression levels or to improve the promoter to compensate for low expression. Most of our assays benefit from high expression levels. Intracellular assays such as the Mito tracker require high levels of expression for visualization of the fluorescent protein in the cell and to sufficiently pull associated proteins to the mitochondria to induce a phenotype. For mass spectrometry or Lumier analysis ([26], high levels of expression are key to reducing the amount of starting material. This reduction decreases cost and increases throughput. In addition, it has been our experience that weak epitope tags (such as a Single-Flag epitope) require the increased expression of our Creator Splice system for routine visualization of proteins by immunoblot analysis (see additional file 5C). The disadvantage of the Creator Splice system is that it requires an additional donor vector and customized acceptor vectors. This disadvantage is relieved to some extent however, because inserts flanked by AscI and PacI sites can be easily swapped between the two donors without having to perform additional DNA sequencing.

## Conclusion

We have modified the Creator system by extending the multiple cloning sites of the donor vectors, creating a repository of downstream vectors, and by increasing expression levels with the Creator Splice system that allows for splicing at the 5' end. Investigators using this system have full control to develop constructs with high expression yet control the tag and linker sequences to maintain or optimize protein function. Our system is designed to facilitate proteomic applications and the determination of protein functions in cell-based model systems. These reagents are available to the scientific community upon request, and it is our hope to build up a set of reagents that can become a strong resource.

## Methods

### Production of Cre recombinase

Cre recombinase was purchased from Clontech or purified in our laboratory as a His-Cre construct (gift from Paul Sadowski [46]). To purify His-Cre, BL21 cells were grown in 600 ml LB broth plus 100 μg/ml ampicillin to an OD_600 _of 0.5, induced with 1 mM IPTG, and allowed to grow for another 4 hours. Cells were pelleted and lysed using a cell homogenizer in lysis buffer (50 mM TrisHCl pH8, 300 mM NaCl, 100 mM PMSF). The lysate was clarified by centrifugation (20,000 × G for 20 minutes) and the supernatant was loaded onto a 5 ml Ni-Nta column (Amersham). The column was washed with 50 mM TrisHCl pH8, 600 mM NaCl, 80 mM Imidazole, and 100 mM PMSF and the protein was eluted in 1 ml fractions using a step-wise gradient starting with wash buffer and ending with elution buffer of 20 mM TrisHCl pH8, 300 mM NaCl, 100 mM PMSF, and 500 mM Imidazole. Positive fractions were pooled, and concentrated using a centrifugal filter (centricon, 5 kDa cutoff, Millipore) into a final solution of 20 mM TrisHCl pH8, 50 mM NaCl, 500 μM EDTA, 1 mM DTT and 50% glycerol.

### Vector construction

Specific details on the cloning of each parent vector are provided in Additional Files 1 (donor vectors) and 2 (acceptor vectors). Sequences of oligonucleotides are provided in Additional File 4. Full sequences of vectors are provided online at the Pawson laboratory website ([47]). Vectors were modified by one of four methods: 1) PCR and restriction enzyme: inserts were amplified by PCR using Expand polymerase (Roche), the insert and vector were digested with the appropriate restriction enzymes, and ligated together using T4 DNA ligase; 2) PCR and recombination: inserts were amplified by PCR using Expand polymerase and recombined into the vector using In Fusion (Clontech) according to the manufacturers instructions; 3) Oligonucleotide hybridization: complementary oligonucleotides (Sigma) were annealed and ligated into digested-vector, 4) Site-directed mutagenesis: mutations were introduced into the vector during PCR amplification of the entire vector using complementary oligonucleotides (Sigma) with the appropriate mutations (Quickchange, Stratagene). To create V678 and V795, the NotI sites in V308 and V309, respectively, were converted to FseI sites by digesting with NotI, filling in overhangs with Klenow DNA polymerase and then re-ligating the vector. All inserts into vectors were sequenced to ensure their integrity. Acceptor vectors were created using Clontech acceptor backbones (pLP ECFP C1 or pLPS-3'EGFP) or by adding the loxP-promoter cassette to pre-existing vectors (Clontech manual PT3576-1). The RFP template for vectors V1662 to V1664 was modified from the Clontech dsRED2 construct using site-directed mutagenesis to incorporate 73 single point mutations (resulting in 36 different amino acids) as outlined [19,18].

### Chemically treated competent cell preparation

Preparation of highly-competent *E. coli *cells was based on the method devised by Inoue [48]. Briefly, 250 ml of cells were grown in SOB media at room temperature until they reach an OD_600 _of 0.6 and then were collected by centrifugation at 2500 × G for 10 minutes at 4°C. The cells were then resuspended in 80 ml of ice-cold TB buffer (10 mM PIPES, 55 mM MnCl_2_, 15 mM CaCl_2_, 250 mM KCl, pH 6.7), incubated on ice for 10 minutes, and the cells were collected once more by centrifugation at 2500 × G for 10 minutes at 4°C. The cells were resuspended in 20 ml ice-cold TB buffer and DMSO was added to a final concentration of 7%. After a further ten minute incubation on ice, the cells were flash-frozen and stored at -80°C.

### ORF cloning and recombination reactions

Additional File 3 outlines details on ORF cloning. Inserts were cloned using DNA ligase or topoisomerase. For ligation reactions, the ORFs were amplified by PCR, digested with AscI and PacI and cloned into AscI/PacI-digested vector. For topoisomerase – inserts were amplified by PCR using Platinum PFx (Invitrogen) and subcloned into a CMV5-based directional TOPO vector with a 5' Triple-Flag sequence ([26]). For recombination reactions, 400 ng of donor and acceptor were recombined in a final volume of 20 μl for 15 minutes at room temperature in the presence of Cre recombinase and either optimized recombinase buffer (5 mM TrisHCl pH7.9, 3.3 mM NaCl, 1 mM MgCl_2 _and 1 mM spermidine) or standard recombinase buffer (50 mM TrisHCl pH7.9, 33 mM NaCl, 10 mM MgCl_2_) according to Clontech manual PT3460-1. Cre recombinase was inactivated by incubation of the reaction at 70°C for 10 minutes. Competent cells (> 1 × 10^8 ^cfu/μg) were transformed with 1 μl of the reaction and the entire transformation was plated onto LB-agar plates containing 30 μg/ml chloramphenicol and 7% sucrose (w/v) to select for the correct recombinant vector. Typical colony numbers for this reaction are between 5 and 100 colonies.

### Immunoblot analysis

HEK293T cells were grown in 6 well dishes in DMEM media supplemented with 10% fetal calf serum (HyClone) and transfected by Polyethylenimine (PEI) using 4 μg of DNA and 10 μg PEI/well [49]. For quantitation of protein expression levels, the cells were co-transfected with V3 (pLP ECFP C1) at 1 μg/well. After 48 hours, the cells were lysed in NP40 lysis buffer (20 mM TrisHCl pH8, 137 mM NaCl, 10% Glycerol, 1% NP40, 10 μg/ml of aprotinin, 10 μg/ml of leupeptin and 1 mM Phenylmethylsulphonylfluoride (PMSF)), and the supernatants clarified by centrifugation at 18,000 × G for 15 minutes. For quantitation of protein expression, the cell lysate was divided between immunoblot analysis and fluorescence analysis. For immunoblot analysis, cell lysates were separated by sodium dodecyl sulphate-polyacrylamide gel electrophoresis (SDS-PAGE), transferred to Polyscreen polyvinylidene fluoride (PVDF) (PerkinElmer), and blocked in tris-buffered saline with 0.05% tween (TBST)-containing 5% skim milk powder. Membranes were probed with the indicated antibodies (0.5 μg/ml M2-Flag (Sigma) or 1 μg/ml 9E10 (anti-Myc, Santa Cruz)) in TBST-containing 5% skim milk powder as previously described [50]. Results were visualized either by exposure to film or with a Fluor-S-Max instrument (BioRad). For the α-Nadrin reprobe, the immunoblot was stripped of antibody by incubating in stripping buffer (150 mM TrisHCl pH6.8, 2% SDS, and 100 mM beta-mercaptoethanol) at 65°C for 20 minutes. The immunoblot was then washed in TBS and blocked in TBST containing 5% skim milk powder. The blot was then reprobed using α-Nadrin antibody (1:1000). Antibodies against Nadrin/Rich1 (#2104) were generated against bacterially expressed His-tagged fusion protein of a.a. 1–245 of human Rich1, in the PET30B expression vector, in Rabbits as described[51]. For quantitation, band intensity, after immunoblot analysis with the appropriate antibody, was determined by the Fluor-S-Max software. To determine fluorescent levels of lysate co-transfected with V3 (pLP ECFP C1), cell lysate was added to a 96 well quartz plate and the lysate was analyzed for fluorescence intensity using a spectrum scan from OD_450 _to OD_520 _with excitation at OD_433_and a cutoff at OD_455 _using a SpectroMax Gemini (Molecular Devices). Subsequent analysis used fluorescent intensity values at OD_480_. To determine the relative expression of each vector, the band intensity of a construct was divided by the relative fluorescent value of the ECFP expression control (V3) and this value was averaged between replicates.

### Immunofluorescence

NIH 3T3 cells (ATCC) were plated on coverslips in 6 well dishes in DMEM media supplemented with 10% fetal calf serum (HyClone). Cells were transfected using lipofectamine 2000 (Invitrogen) according to the manufacturers protocol. After 24 hours, cells were fixed in 4% paraformaldehyde in phosphate-buffered saline (PBS) for ten minutes and then permeabilized in 0.3 % Triton X-100 for five minutes. Cells were then incubated overnight with a mixture of primary antibodies containing rabbit-anti-GFP (AbCAM) at 1:200 dilution and mouse-anti-Cytochrome C (BD Pharmingen) at 0.1 μg/ml. Samples were extensively rinsed with PBS and subsequently labeled with a mixture of secondary antibodies containing goat-anti-mouse Texas Red and goat-anti-rabbit Alexa 488 at 20 μg/ml (Molecular Probes) for one hour. After incubation, and rinses with PBS, Hoechst 33258dye (Molecular Probes) was added at a 1:1000 dilution to label nuclei and slides were mounted in Geltol (Fisher Scientific). Samples were then imaged on a Deltavision Deconvolution Microscope (Applied Precision Inc., Issaquah, WA) using an Olympus 100× (N.A. 1.35) objective lens and raw images were subsequently deconvolved.

## Authors' contributions

KC designed and created most of the donor and acceptor vectors discussed, performed immunoblot analysis and drafted the manuscript, CDW aided in vector design and protocol development and aided in drafting the manuscript, KE and KH aided in vector construction and performed immunoblot analysis, MG created most of the expression vectors, SK performed the immunofluorescence analysis, WRH performed mutagenesis on the dsRED2 constructs, TP participated in the design of the study and aided in drafting the manuscript, GBM conceived of the study and participated in its design and helped to draft the manuscript. All authors read and approved the final manuscript.

## Supplementary Material

Additional File 1 Table: construction of donor vectors – provides details on the reagents used to create new donors Click here for file

Additional File 2 Table: construction of acceptor vectors – provides details on the reagents used to create new acceptors Click here for file

Additional File 4 Table: oligonucleotide sequences – provides sequences of oligonucleotides used to clone donor, acceptor and expression vectors Click here for file

Additional File 5 Figure: expression levels of various expression vectors – provides gel or immunoblot images of various expression vectors that have been tested Click here for file

Additional File 3 Table: construction of expression vectors – provides details on ORF sources and vectors used for construction Click here for file

## Figures and Tables

**Table 1:** donor vectors constructs

***Vector ID***	***Vector Name***	***Potential Splicing Events***	***Description***
V1	pDNR Dual	Splice for 3' tags	Designed for Fusions at both N- and C- termini (Clontech)
V7	pDNR-MCS	Splice for 3' tags	Alternate MCS that adds AscI and PacI sites to the MCS of V1
V37	pDNR MCS SA	Splice for 5' and 3' tags	A splice acceptor introduced into V7 before the AscI site
V308	pDNR MCS lacZ alpha	Splice for 3' tags	LacZ alpha inserted between AscI and PacI sites of V7
V309	pDNR MCS SA lacZ alpha	Splice for 5' and 3' tags	LacZ alpha inserted between AscI and PacI sites of V37
V624	pDNR MCS BE	Splice for 3' tags	Alternate MCS with BamHI, SalI, SmaI, HindIII, EcoRI sites
V677	pDNR MCS BE lacZ alpha	Splice for 3' tags	LacZ alpha inserted between SalI and HindII sites of V624
V678	pDNR MCS SA ES	Splice for 5' and 3' tags	Alternate MCS Splice Acceptor with AscI, EcoRI, NotI, BamHI, SalI, PacI sites.
V795	pDNR MCS ES	Splice for 3' tags	Alternate MCS with AscI, EcoRI, NotI, BamHI, SalI, PacI sites
V954	pDNR MCS SA no SA CmR	Splice for 5' and 3' tags	Silent mutations introduced in the CmR ORF of V309 to remove potential splice acceptor

*Abbreviations used: SA – splice acceptor, MCS – multiple cloning site, CmR – chloramphenicol resistance, BE – BamHI/EcoRI, ES – EcoRI/SalI

**Table 2:** acceptor vectors constructs

***Vector ID***	***Vector Name***	***Potential Splicing Events***	***Tag***	***Tag Position***	***Promoter***	***Function***	***Expression Verification***
V3	pLP-ECFP C1 (Clontech)*	none	ECFP	N-terminus	CMV	localization	Verified by Clontech
V4	pLP-EGFP C1 (Clontech)	none	EGFP	N-terminus	CMV	localization	Verified by Clontech
V5	pLP-EYFP C1 (Clontech)	none	EYFP	N-terminus	CMV	localization	Verified by Clontech
V6	pLPS-3'EGFP (Clontech)	Splice for 3' Tags	EGFP	C-terminus	CMV	localization	Verified by Clontech
V956	pLPS-3'ECFP	Splice for 3' Tags	ECFP	C-terminus	CMV	localization	Figure 3
V957	pLPS-3'ECFP-mito	Splice for 3' Tags	ECFP-mito	C-terminus	CMV	targets to mitochondria	Figure 3
V1662	pLP-RFP	none	RFP	N-terminus	CMV	localization	Additional File 5A
V1663	pLPS-3'RFP	Splice for 3' Tags	RFP	C-terminus	CMV	localization	Verified, Data not Shown
V1664	pLPS-3'RFP-mito	Splice for 3' Tags	RFP-mito	C-terminus	CMV	targets to mitochondria	Verified, Data not Shown
V25	pLP Flag	none	Flag	N-terminus	CMV	IP/IB/IF	Additional File 5C
V26	pLPS-3'Flag	Splice for 3' Tags	Flag	C-terminus	CMV	IP/IB/IF	Additional File 5B
V33	pLP Flag SD	Splice for 5' Tags	Flag	N-terminus	CMV	IP/IB/IF	Additional File 5C
V179	pLP Triple-Flag	none	Triple-Flag	N-terminus	CMV	IP/IB/IF	Figure 4&5
V180	pLP Triple-Flag SD	Splice for 5' Tags	Triple-Flag	N-terminus	CMV	IP/IB/IF	Figure 4&5
V181	pLPS 3' Triple-Flag	Splice for 3' Tags	Triple-Flag	C-terminus	CMV	IP/IB/IF	Figure 4&5
V207	pRETRO-Triple-Flag SD	Splice for 5' Tags	Triple-Flag	N-terminus	LTR	IP/IB/IF/retrovirus^%^	Additional File 5D
V143	pLP-HA SD	Splice for 5' Tags	HA	N-terminus	CMV	IP/IB/IF	Additional File 5E
V27	pLPS-3'HA	Splice for 3' Tags	HA	C-terminus	CMV	IP/IB/IF	Not expression verified
V516	pLP-dMyc	none	Double-Myc	N-terminus	CMV	IP/IB/IF	Figure 4
V517	pLP-dMyc SD	Splice for 5' Tags	Double-Myc	N-terminus	CMV	IP/IB/IF	Figure 4
V518	pLPS-3'dMyc	Splice for 3' Tags	Double-Myc	C-terminus	CMV	IP/IB/IF	Figure 4
V621	pGEX-4T-LP	none	GST	N-terminus	Tac	Bacteria expression	Verified, Data not Shown
V622	pGEX-2TK-LP	none	GST	N-terminus	Tac	Bacteria expression	Additional File 5F
V623	pAcGHLT-LP	none	GST His	N-terminus	Ph	Baculovirus expression	Not expression verified
V1579	pLP ProHis HTb	none	His	N-terminus	Trc	Bacteria expression	Additional File 5G

*Abbreviations used: LP – loxP, mito – mitochondria localization peptide, SD – splice donor, IP – immunoprecipitation, IB – immunoblot analysis, IF – immunofluorescence, Ph – polyhedrin

^%^V207 has been used extensively to produce protein from transient or stably transfected cells. It has not been tested for retrovirus production or resulting protein expression from a retrovirus.
