# Supplementary material for: Modification of the Creator recombination system for proteomics applications – improved expression by addition of splice sites
Source: BMC Biotechnol. 2006 Mar 6;6:13. doi: 10.1186/1472-6750-6-13 (PMC1421398; doi:10.1186/1472-6750-6-13)
Supplement: Additional File 1 — Table: construction of donor vectors – provides details on the reagents used to create new donors [file 1472-6750-6-13-S1.pdf]

Additional Table 1) Construction of Donor Vectors

Vector ID	***Vector Name***	***Oligos Used for Cloning and Method***	***Vector and Cloning Sites***
V1	pDNR Dual	Clontech Vector	Clontech Vector
V7	pDNR-MCS	O1 & O2 – PCR using V1 as template	BglII/AvrII-digested V1
V37	pDNR MCS SA	O3 & O4 - hybridization	AscI-digested V7
V308	pDNR MCS LacZ alpha	O78 & O79 – PCR using LacZ alpha template from Yunping Lin	AscI/PacI-digested V7
V309	pDNR MCS SA LacZ alpha	O78 & O79 – PCR using LacZ alpha template from Yunping Lin	AscI/PacI-digested V37
V624	pDNR MCS BE	O110 & O111 - hybridization	NheI/AvrII-digested V1
V677	pDNR MCS BE LacZ alpha	LacZ alpha template - gift of Yunping Lin	SalI/HindIII-digested V624
V678	pDNR MCS SA ES	O374 & O375 - hybridization	AscI/PacI-digested V309 with NotI site mutated
V795	pDNR MCS ES	O374 & O375 - hybridization	AscI/PacI-digested V308 with NotI site mutated
V954	pDNR MCS SA no SA CmR	O384 & O385 – mutagenesis	Mutagenesis of V309
