# Supplementary material for: Modification of the Creator recombination system for proteomics applications – improved expression by addition of splice sites
Source: BMC Biotechnol. 2006 Mar 6;6:13. doi: 10.1186/1472-6750-6-13 (PMC1421398; doi:10.1186/1472-6750-6-13)
Supplement: Additional File 2 — Table: construction of acceptor vectors – provides details on the reagents used to create new acceptors [file 1472-6750-6-13-S2.pdf]

# Additional Table 2) Construction of Acceptor Vectors

| *Vector ID* | *Vector Name* | *Oligos Used for Cloning and Method* | *Vector and Cloning Sites* |
|---|---|---|---|
| V3 | pLP-ECFP C1 (Clontech)* | Clontech Vector | Clontech Vector |
| V4 | pLP-EGFP C1 (Clontech) | Clontech Vector | Clontech Vector |
| V5 | pLP-EYFP C1 (Clontech) | Clontech Vector | Clontech Vector |
| V6 | pLPS-3'EGFP (Clontech) | Clontech Vector | Clontech Vector |
| V956 | pLPS-3'ECFP | O388 & O389 – PCR using V3 as template | AgeI/XbaI-digested V6 |
| V957 | pLPS-3'ECFP-mito | O390 & O391 – PCR using pSPL35 as template | BsrGI/MfeI-digested V956 |
| V1662 | pLP-RFP | O484 & O485 – PCR using mutated dsRED2 as template | NheI/HindIII-digested V3 |
| V1663 | pLPS-RFP | O486 & O487 – PCR using mutated dsRED2 as template | AgeI/XbaI-digested V6 |
| V1664 | pLPS-3'RFP-mito | O486 & O488 – PCR using mutated dsRED2 as template | AgeI/BsrGI-digested V957 |
| V25 | pLP Flag | O5 & O6 - hybridization | NheI/HindIII-digested V3 |
| V26 | pLPS-3'Flag | O15 & O16 - hybridization | AgeI/XbaI-digested V6 |
| V24 | pLP-ECFP Intron | O7 & O8 – PCR using accession J01917 as template | BglII/HindIII-digested V3 by In Fusion |
| V33 | pLP Flag SD | O9 & O10 - hybridization | NheI/BglII-digested V24 |
| V179 | pLP Triple-Flag | O28 & O29 - hybridization | NheI/HindIII-digested V3 |
| V180 | pLP Triple-Flag SD | O30 & O31 - hybridization | NheI/BglII-digested V24 |
| V181 | pLPS 3' Triple Flag | O32 & O33 - hybridization | AgeI/XbaI-digested V6 |
| V207 | pRETRO-Triple-Flag SD | O74 & O75 – PCR using V180 as template | XhoI/HpaI-digested pMSCV puro (Clontech) |
| V143 | pLP-HA SD | O13 & O14 - hybridization | NheI/BglII-digested V24 |
| V27 | pLPS-3'HA | O17 & O18 - hybridization | AgeI/XbaI-digested V6 |
| V516 | pLP-dMyc | O82 & O83 - hybridization | NheI/HindIII-digested V3 |
| V517 | pLP-dMyc SD | O84 & O85 - hybridization | NheI/BglII-digested V24 |
| V518 | pLPS-3'dMyc | O86 & O87 - hybridization | AgeI/XbaI-digested V6 |
| V621 | pGEX-4T-LP | O107 & O108 – PCR using V3 as template | BamHI/EcoRI-digested pGEX 4T-2 (Amersham) |
| V622 | pGEX-2TK-LP | O107 & O108 – PCR using V3 as template | BamHI/EcoRI-digested pGEX 2TK (Amersham) |
| V623 | pAcGHLT-LP | O109 & O108 – PCR using V3 as template | NdeI/EcoRI-digested pAc GHLT C (Pharmingen) |
| V1579 | pLP-ProEX HTb | O107 & O108 – PCR using V3 as template | BamHI/EcoRI-digested pProEX HTb (Invitrogen) |

*Abbreviations used: LP – loxP, SD – splice donor, mito – mitochondria localization signal
