# Supplementary material for: Modification of the Creator recombination system for proteomics applications – improved expression by addition of splice sites
Source: BMC Biotechnol. 2006 Mar 6;6:13. doi: 10.1186/1472-6750-6-13 (PMC1421398; doi:10.1186/1472-6750-6-13)
Supplement: Additional File 4 — Table: oligonucleotide sequences – provides sequences of oligonucleotides used to clone donor, acceptor and expression vectors [file 1472-6750-6-13-S4.pdf]

Additional Table 4) Oligonucleotides Sequences

Oligo ID	*Oligo Sequence*
O1	CATTAGATCTATAACTTCGTATAGCATACATTATACGAAGTTA TGGCGCGCCGTC GACGGTACCGGACATATGCCCGGGAAT
O2	ATCCCTAGGAACTTACCTGGTTAATTAAGGCCCGCGCGC AAACGAATGGTC T
O3	CGCGAGGGTTTCCTTGACAATATCATACTTATCCTGTCCCTTTTTTTTCCCCAGGGG
O4	CGCGCCCCTGGGGAAAAAAAAGGGACAGGATAAGTATGATATTGTCAAGGAAACCCT
O5	CTAGCCACCATGGACTACAAGGACGACGACGACAAGCA
O6	AGCTTGCTTGTCGTCGTCGTCCTTGTAGTCCATGGTGG
O7	TCCGGACTCAGATCTTGACCTCTCAAAAGCGGGCATG
O8	AAGTTATCGAAGCTTAGCGTTGACCTTGTCACCCT
O9	CTAGCCACCATGGACTACAAGGACGACGACGACAAGGTAAGTA
O10	GATCTACTTACCTTGTCGTCGTCGTCCTTGTAGTCCATGGTGG
O13	CTAGCCACC ATG TAC CCA TAC GAC GTG CCA GAC TAC GCC CAG GTAAGTA
O14	GATCTACTTACCTGGGCGTAGTCTGGCACGTCGTATGGGTACATGGTGG
O15	CCG GTG GAC TAC AAG GAC GAC GAC GAC AAG TAA T
O16	CTAGATTACTTGTCGTCGTCGTCCTTGTAGTCCA
O17	CCG GTG TAC CCA TAC GAC GTG CCA GAC TAC GCC TAA T
O18	CTAGATTAGGCGTAGTCTGGCACGTCGTATGGGTACA
O28	CTAGCCACCATGGACTACAAGGACCACGACGGCGACTACAAGGACCACGACATCGACTACAAGGACGACGACGACAAGCA
O29	AGCTTGCTTGTCGTCGTCGTCCTTGTAGTCGATGTCGTGGTCCTTGTAGTCGCCGTCGTGGTCCTTGTAGTCCATGGTGG
O30	CTAGCCACCATGGACTACAAGGACCACGACGGCGACTACAAGGACCACGACATCGACTACAAGGACGACGACGACAAGGTAAGTA
O31	GATCTACTTACCTTGTCGTCGTCGTCCTTGTAGTCGATGTCGTGGTCCTTGTAGTCGCCGTCGTGGTCCTTGTAGTCCATGGTGG
O32	CCGGTGGACTACAAGGACCACGACGGCGACTACAAGGACCACGACATCGACTACAAGGACGACGACGACAAGTAAT
O33	CTAGATTACTTGTCGTCGTCGTCCTTGTAGTCGATGTCGTGGTCCTTGTAGTCGCCGTCGTGGTCCTTGTAGTCCA
O74	TACGTCTCGAGCCACCATGGACTACAAGGACC
O75	TACGTGTTAACACGTCAGGTGGCACTTTTCG
O78	TCGAGGCGCGCCGGCGCCCAATACGCAAAC
O79	GCCATTAATTAACGCCATTCGCCATTCAGG
O82	CTAGCCACCATGGAGCAGAAGCTGATCAGCGAGGAGGACCTGGGCGGCGAGCAGAAGCTGATCAGCGAGGAGGACCTGCA
O83	AGCTTGCAGGTCCTCCTCGCTGATCAGCTTCTGCTCGCCGCCCAGGTCCTCCTCGCTGATCAGCTTCTGCTCCATGGTGG
O84	CTAGCCACCATGGAGCAGAAGCTGATCAGCGAGGAGGACCTGGGCGGCGAGCAGAAGCTGATCAGCGAGGAGGACCTGGGCGGCCAGGTAAGTA
O85	GATCTACTTACCTGGCCGCCCAGGTCCTCCTCGCTGATCAGCTTCTGCTCGCCGCCCAGGTCCTCCTCGCTGATCAGCTTCTGCTCCATGGTGG
O86	CCGGTGGAGCAGAAGCTGATCAGCGAGGAGGACCTGGGCGGCGAGCAGAAGCTGATCAGCGAGGAGGACCTGTAAT
O87	CTAGATTACAGGTCCTCCTCGCTGATCAGCTTCTGCTCGCCGCCCAGGTCCTCCTCGCTGATCAGCTTCTGCTCCA
O95	ATCGGGCGCGCCATGGCATCTGCCAGCTCTAGCCGGGCAGGA
O96	ATCGTTAATTAACTCCTGTCCAAAGCCCTCGGTCTCCTCAAT
O103	ATCGGGCGCGCCATGGCGTCTCCTTCTAGAAACTCCCAGAGC
O104	ATCGTTAATTAACTCAAGTCCAAAGGTGCTGGTTTCCTCTAC
O107	TACGGGATCCATAACTTCGTATAGCATACA

O108	CGTCGAATTCACGTCAGGTGGCACTT
O109	TGCGCATATGGATAACTTCGTATAGCATACA
O110	CT AGC ATA ACT TCG TAT AGC ATA CAT TAT ACG AAG TTA TGG GGA TCC GTC GAC CCC GGG AAG CTT GAA TTC CAG GTA AGT TAG C
O111	CT AGG CTA ACT TAC CTG GAA TTC AAG CTT CCC GGG GTC GAC GGA TCC CCA TAA CTT CGT ATA ATG TAT GCT ATA CGA AGT TAT G
O277	CTA GGA ACT TAC CTG GTT AAT TAA TGA AAC GTT GGT GGG CTG
O301	TTC CCC AGG GGC GCG CCA TGC CTC GGG CTC AGC CAT C
O374	CGCGCCG GGAATTCAGC GGCCGCGGGA TCCGTCGACT TAAT
O375	GGC CCTTAAGTCG CCGGCGCCCT AGGCAGCTGA AT
O384	TCT CCA TTT TGG CTT CCT TGG CTC CTG AAA GAT CC
O385	GGA TCT TTC AGG AGC CAA GGA AGC CAA AAT GGA GA
O388	GTACACCGGTGAGCAAGGGCGAGGAGCTG
O389	GATCTCTAGATTACTTGTACAGCTCGTCTACGC
O390	GATCTGTACAAGAAACTAATTGCTAAAAG
O391	ATGACAATTGTTAATTATTTTTTCTTAATTG
O484	CTAG GCTAGC CCACC ATG GCC TCC TCC GAG GAC GTC ATC AAG
O485	CTAG AAGCTT GG GCG CCG GTG GAG TGG CGG CCC TCG GC
O486	CTAG ACCGGT G GCC TCC TCC GAG GAC GTC ATC AAG
O487	CTAG TCTAGA TTA GGC GCC GGT GGA GTG GCG GCC CTC GGC
O488	CTAG TGTACA G GGC GCC GGT GGA GTG GCG GCC CTC GGC
O519	AAG TTA TGG CGC GCC ATG GGA GAA GTC GCC G
O520	TAC CTG GTT AAT TAA TGA AAG GCC TGT CTG AG
O533	AAG TTA TGG CGC GCC ATG CTA TCC AGC CGG TG
O535	TAC CTG GTT AAT TAA GGG GAA GAG GCT GGT G
O541	AAG TTA TGG CGC GCC ATG AAG AAG CAA TTC AAC CG
O547	TAC CTG GTT AAT TAA TAC ATC AGT GAC GAT TCT GG
O588	AAG TTA TGG CGC GCC ATG ACA GCC AAT CAT GAG AG
O589	AAG TTA TGG CGC GCC ATG AGA CTT GAT GTG AAC TT
O594	TAC CTG GTT AAT TAA CTG GAT CCA GAT TGT GTT TC
O595	TAC CTG GTT AAT TAA GAT TTT TGT TTC TGG ACC CT
O714	AAG TTA TGG CGC GCC ATG ACG TTG CTG ATC ACT G
O720	TAC CTG GTT AAT TAA TTT TTT GAA GGG GGT TAA CC
O926	CACCATGACGTTGCTGATCACTG
O927	GAATTCATCATTTTTTGAAGGGGGTTAAC
O928	CAC CAT GAC AGC CAA TCA TGA GAG
O929	GAA TTC ATC ACT GGA TCC AGA TTG TG
O930	CAC CAT GAA GAA GCA ATT CAA CCG
O931	GAA TTC ATC ATA CAT CAG TGA CGA TTC
O932	CAC CAT GCT ATC CAG CCG GTG
O933	GAA TTC ATC AGG GGA AGA GGC TG
O934	CAC CAT GGG AGA AGT CGC CG
O935	GAA TTC ATC ATG AAA GGC CTG TCT G

O936	CAC CAT GAG ACT TGA TGT GAA CTT C
O937	GAA TTC ATT AGA TTT TTG TTT CTG GAC C
