# Supplementary material for: Modification of the Creator recombination system for proteomics applications – improved expression by addition of splice sites
Source: BMC Biotechnol. 2006 Mar 6;6:13. doi: 10.1186/1472-6750-6-13 (PMC1421398; doi:10.1186/1472-6750-6-13)
Supplement: Additional File 5 — Figure: expression levels of various expression vectors – provides gel or immunoblot images of various expression vectors that have been tested [file 1472-6750-6-13-S5.pdf]

Additional File 5 Colwill et al.

A) Expression Check for Creator Vector V1662 - pLP RFP

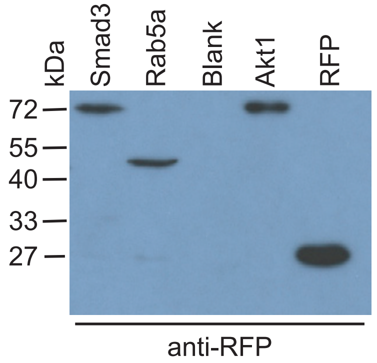

HEK293T cells were transfected with the indicated constructs using PEI. After 48 hours, the cells were harvested in PLC lysis buffer and the lysate was clarified by centrifugation. The lysate was resolved by SDS-PAGE and immunoblot analysis was performed using anti-RFP antibodies (Chemicon,1:1000). Abbreviations: kDa - kilodaltons. Cloning details provided in additional table 3b.

B) Expression Check for Creator Vector V26 - pLPS-3'Flag

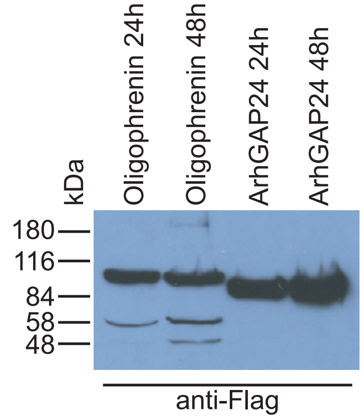

HEK293T cells were transfected with the indicated constructs using CaP04. After the indicated times, the cells were harvested in NP40 lysis buffer and the lysate was clarified by centrifugation. The lysate was resolved by SDS-PAGE and immunoblot analysis was performed using anti-M2 Flag antibodies (Sigma,0.5 µg/ml). Abbreviations: kDa - kilodaltons. Cloning details provided in additional table 3b.

C) Expression Check for Creator Vectors V25 and V33

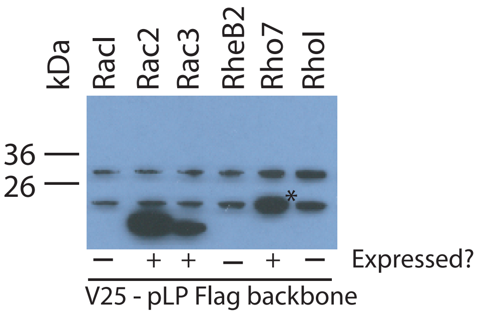

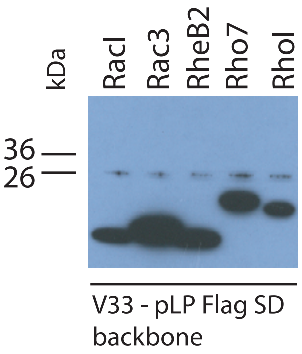

HEK293T cells were transfected with the indicated constructs using PEI. After 24 hours, the cells were harvested in PLC lysis buffer and the lysate was clarified by centrifugation. The lysate was resolved by SDS-PAGE and immunoblot analysis was performed using anti-M2 Flag antibodies (Sigma, 1:1000). The asterick marks the expression of Rho7 in the V25 backbone. A subset of the ORFs in V25 were also expressed in V33. Note that all ORFs from the V33 backbone tested positive for expression (with splicing) whereas only half of the constructs from the V25 backbone (no splicing) tested positive. Abbreviations: kDa - kilodaltons. Cloning details provided in additional table 3b.

D) Expression Check for Creator Vector V207 - pRETRO Triple Flag SD

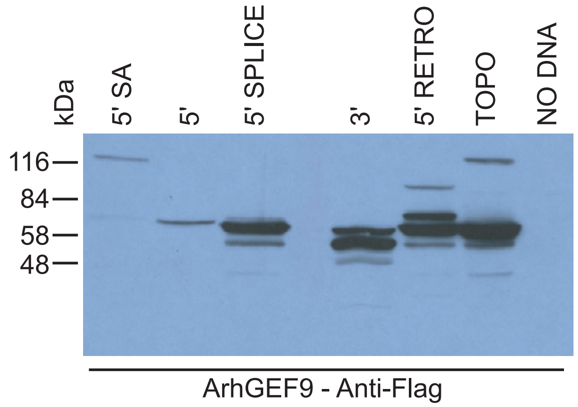

HEK293T cells were transfected with the indicated constructs using CaPO4. After 48 hours, the cells were harvested in NP40 lysis buffer and the lysate was clarified by centrifugation. The lysate was resolved by SDS-PAGE and immunoblot analysis was performed using anti-M2 Flag antibodies (Sigma,0.3 μg/ml). Abbreviations: kDa - kilodaltons, 5' SA -Creator Splice donor vector with Creator 5' tag acceptor vector (no splicing event), 5' - Creator 5' tag, 5' SPLICE - Creator 5' tag with splicing, 3' - Creator 3' tag acceptor vectors, 5' RETRO - V207 as acceptor, with splicing, TOPO - 5' tag, non-Creator expression system. Cloning details provided in additional table 3b.

E) Expression Check for Creator Vector V143 - pLP HA SD

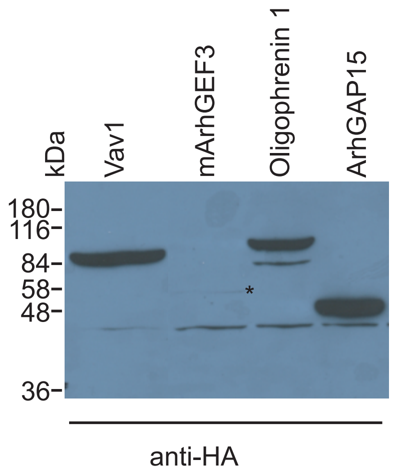

HEK293T cells were transfected with the indicated constructs using CaP04. After 24 hours, the cells were harvested in NP40 lysis buffer and the lysate was clarified by centrifugation. The lysate was resolved by SDS-PAGE and immunoblot analysis was performed using anti-HA antibodies (1:1000). The asterick in lane 2 indicates HA-tagged mArhGEF3. Abbreviations: kDa - kilodaltons. Cloning details provided in additional table 3b.

F) Expression Check for Creator Vector V622 - pGEX 2TK LP

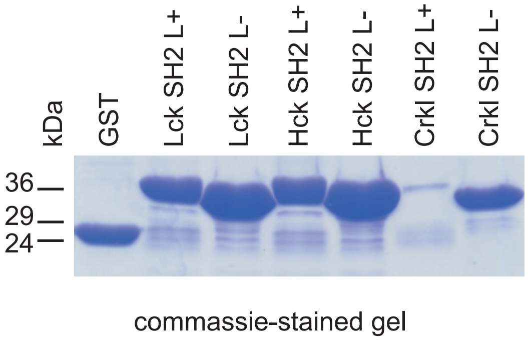

Three ml cultures of *E. coli* expressing vectors for the indicated constructs were induced with 1 mM IPTG at an OD_{600} of 0.6. The cells were lysed into GST-binding buffer and the lysate was clarified by centrifugation. GST fusions were purified using glutathione agarose (Amersham) and the protein was released by boiling in SDS-sample buffer. The eluate was resolved by SDS-PAGE and the gel was stained with commassie blue. Abbreviations: kDa - kilodalton, L- lacks loxP site pGEX-2TK-based, L+ contains loxP site, V622-based. Cloning details provided in additional table 3b.

G) Expression Check for Creator Vector V1579 - pLP ProEx HTb

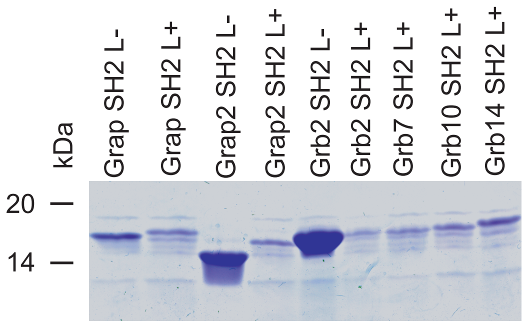

commasie-stained gel

Three ml cultures of *E. coli* expressing vectors for the indicated constructs was induced with 0.5 mM IPTG at an OD_{600} of 0.6. The cells were lysed into His-binding buffer and the lysate was clarified by centrifugation. His fusions were purified using Talon metal affinitiy resin (Clontech) and the protein was released by boiling in SDS-sample buffer. The eluate was resolved by SDS-PAGE and the gel was stained with commassie blue. Abbreviations: kDa - kilodalton, L- lacks loxP site pProEx HTb-based, L+ contains loxP site, V1579-based. Cloning details provided in additional table 3b.

Lysis Buffers:

NP40 Lysis Buffer -20 mM TrisHCl pH8, 137 mM NaCl, 10% Glycerol, 1% NP40, 10 μg/ml of aprotinin, 10 μg/ml of leupeptin and1 mM Phenylmethylsulphonylfluoride (PMSF))

PLC Lysis Buffer - 50 mM Hepes pH 7.5, 150 mM NaCl, 3% glycerol, 1% Triton X-100, 1.5 mM $MgCl_2$, 1 mM EGTA, 10 mM sodium pyrophosphate, 100 mM NaF, 10 μg/ml of aprotinin, 10 μg/ml of leupeptin and 1 mM Phenylmethylsulphonylfluoride (PMSF))

GST Binding Buffer - PBS with 1% Triton X-100 and 1 mM PMSF

His Binding Buffer - 20 mM TrisHCl pH 7.9, 50 mM NaCl, 5 mM Imidazole, 1% Triton X-100, 1 mM PMSF
