# Supplementary material for: Modification of the Creator recombination system for proteomics applications – improved expression by addition of splice sites
Source: BMC Biotechnol. 2006 Mar 6;6:13. doi: 10.1186/1472-6750-6-13 (PMC1421398; doi:10.1186/1472-6750-6-13)
Supplement: Additional File 3 — Table: construction of expression vectors – provides details on ORF sources and vectors used for construction [file 1472-6750-6-13-S3.pdf]

Additional Table 3a) Construction of Expression Vectors

Vector ID	*Source of Insert*	*Donor Vector*	*Acceptor Vector*	*Name*
V310	I233 = O519 & O520 – PCR with BC012488 as template	V50 = I233 + AscI/PacI-digested V7	V179	5’ Triple-Flag ArhGEF1
V311	I233 = O519 & O520 – PCR with BC012488 as template	V50 = I233 + AscI/PacI-digested V7	V181	3’Triple-Flag ArhGEF1
V312	I233 = O519 & O520 – PCR with BC012488 as template	V91 = I233 + AscI/PacI-digested V37	V180	SPLICE Triple-Flag ArhGEF1
V313	I234 = O541 & O547 – PCR with BC003259 as template	V41 = I234 + AscI/PacI-digested V7	V179	5’ Triple-Flag Nadrin
V314	I234 = O541 & O547 – PCR with BC003259 as template	V41 = I234 + AscI/PacI-digested V7	V181	3’ Triple-Flag Nadrin
V315	I234 = O541 & O547 – PCR with BC003259 as template	V222 = I234 + AscI/PacI-digested V37	V180	SPLICE Triple-Flag Nadrin
V316	I235 = O533 & O535 – PCR with BC006107 as template	V47 = I235 + AscI/PacI-digested V7	V179	5’ Triple-Flag ArhGAP9
V317	I235 = O533 & O535 – PCR with BC006107 as template	V47 = I235 + AscI/PacI-digested V7	V181	3’ Triple-Flag ArhGAP9
V318	I235 = O533 & O535 – PCR with BC006107 as template	V100 = I235 + AscI/PacI-digested V37	V180	SPLICE Triple-Flag ArhGAP9
V319	I231 = O714 & O720 – PCR with AB007884 as template	V117 = I231 +AscI/PacI-digested V7	V179	5’ Triple-Flag ArhGEF9
V320	I231 = O714 & O720 – PCR with AB007884 as template	V117 = I231 +AscI/PacI-digested V7	V181	3’ Triple-Flag ArhGEF9
V321	I231 = O714 & O720 – PCR with AB007884 as template	V210 = I231 +AscI/PacI-digested V37	V180	SPLICE Triple-Flag ArhGEF9
V322	I230 = O588 & O594 – PCR with BC0027070 as template	V54 = I230 +AscI/PacI-digested V7	V179	5’ Triple-Flag ArhGap24
V323	I230 = O588 & O594 – PCR with BC0027070 as template	V54 = I230 +AscI/PacI-digested V7	V181	3’Triple-Flag ArhGap24
V324	I230 = O588 & O594 – PCR with BC0027070 as template	V97 = I230 + AscI/PacI-digested V37	V180	SPLICE Triple-Flag ArhGap24
V325	I232 = O589 & O595 – PCR with BC027830 as template	V48 = I232 + AscI/PacI-digested V7	V179	5’ Triple-Flag StarD13
V326	I232 = O589 & O595 – PCR with BC027830 as template	V48 = I232 + AscI/PacI-digested V7	V181	3’ Triple-Flag StarD13
V327	I232 = O589 & O595 – PCR with BC027830 as template	V103 = I232 + AscI/PacI-digested V37	V180	SPLICE Triple-Flag StarD13

V430	I233 = O519 & O520 – PCR with BC012488 as template	V91 = I233 + AscI/PacI-digested V37	V179	5’ SA Triple-Flag ArhGEF1
V433	I231 = O714 & O720 – PCR with AB007884 as template	V210 = I231 +AscI/PacI-digested V37	V179	5’ SA Triple-Flag ArhGEF9
V436	I233 = O519 & O520 – PCR with BC012488 as template	V91 = I233 + AscI/PacI-digested V37	V181	3’ SA Triple-Flag ArhGEF1
V439	I231 = O714 & O720 – PCR with AB007884 as template	V210 = I231 +AscI/PacI-digested V37	V181	3’ SA Triple-Flag ArhGEF9
V626	I125 = O95 & O96 – PCR with BC013645 as template	V592 = I125 + AscI/PacI-digested V308	V516	5’ Double-Myc WWP2
V627	I127 = O103 & O104 – PCR with AB002320 as template	V593 = I127 + AscI/PacI-digested V308	V516	5’ Double-Myc BulI
V628	I125 = O95 & O96 – PCR with BC013645 as template	V594 = I125 + AscI/PacI-digested V309	V517	SPLICE double-Myc WWP2
V629	I127 = O103 & O104 – PCR with AB002320 as template	V595 = I127 + AscI/PacI-digested V309	V517	SPLICE double-Myc BulI
V630	I125 = O95 & O96 – PCR with BC013645 as template	V592 = I125 + AscI/PacI-digested V308	V518	3’ Double-Myc WWP2
V631	I127 = O103 & O104 – PCR with AB002320 as template	V593 = I127 + AscI/PacI-digested V308	V518	3’ Double-Myc BulI
V1110	I51 = O301 & O277 – PCR with AB028994 as template	V690 = I51 + AscI/PacI-digested V308	V957	3’ Angiomotin N-terminus-ECFP-mito
V1113	I51 = O301 & O277 – PCR with AB028994 as template	V690 = I51 + AscI/PacI-digested V308	V956	3’ Angiomotin N-terminus-ECFP-mito
V1840	I485 = O926 & O927 – PCR with AB007884 as template	I485 inserted into topo-activated pCMV-Triple Flag vector	N/A	Topo Triple-Flag ArhGEF9
V1841	I486 = O928 & O929 – PCR with BC0027070 as template	I486 inserted into topo-activated pCMV-Triple Flag vector	N/A	Topo Triple-Flag ArhGap24
V1842	I487 = O930 & O931 – PCR with BC003259 as template	I487 inserted into topo-activated pCMV-Triple Flag vector	N/A	Topo Triple-Flag Nadrin
V1843	I488 = O934 & O935 – PCR with BC027830 as template	I488 inserted into topo-activated pCMV-Triple Flag vector	N/A	Topo Triple-Flag ArhGEF1
V1844	I489 = O936 & O937 – PCR with BC012488 as template	I489 inserted into topo-activated pCMV-Triple Flag vector	N/A	Topo Triple-Flag StarD13
V1845	I490 = O932 & O933 – PCR with BC006107 as template	I490 inserted into topo-activated pCMV-Triple Flag vector	N/A	Topo Triple-Flag ArhGAP9

Additional Table 3b) Construction of Expression Vectors used in Additional File 5

Vector ID	Source of Insert	Donor Vector Backbone	Acceptor Vector	Name
V86	BC004845 ORF	V7	V26	3’ Flag-Oligophrenin 1
V88	BC027070 ORF	V7	V26	3’ Flag-ArhGAP24
V144	BC013361 ORF	V37	V143	SPLICE HA-Vav1
V145	BC012262 ORF	V37	V143	SPLICE HA-ArhGEF3
V146	BC004845 ORF	V37	V143	SPLICE HA-Oligophrenin 1
V147	BC034881 ORF	V37	V143	SPLICE HA-ArhGAP15
V331	AB007884 ORF	V37	V207	5’ RETRO Triple-Flag ArhGEF9
V468	BC004247 ORF	V7	V25	5’ Flag-RacI
V469	NM_002872 ORF	V7	V25	5’ Flag-Rac2
V470	NM_005052 ORF	V7	V25	5’ Flag-Rac3
V471	BC016155 ORF	V7	V25	5’ Flag RheB2
V472	BC018096 ORF	V7	V25	5’ Flag Rho7
V473	BC005362 ORF	V7	V25	5’ Flag RhoI
V525	BC004247 ORF	V309	V33	SPLICE Flag RacI
V526	NM_005052 ORF	V309	V33	SPLICE Flag Rac3
V527	BC016155 ORF	V309	V33	SPLICE Flag RheB2
V528	BC018096 ORF	V309	V33	SPLICE Flag Rho7
V529	BC005362 ORF	V309	V33	SPLICE Flag RhoI
V936	BC043500 SH2	V624	V622	GST-loxP-Crkl SH2
V939	BC014435 SH2	V624	V622	GST-loxP-Hck SH2
V941	BC013200 SH2	V624	V622	GST-loxP-Lck SH2
V1461	BC014435 SH2	n/a	pGEX 2TK	GST-Hck SH2
V1462	BC013200 SH2	n/a	pGEX 2TK	GST-Lck SH2
V1463	BC043500 SH2	n/a	pGEX 2TK	GST-Crkl SH2
V1575	BC063035 SH2	n/a	pPRO Ex HTb	His-Grap SH2
V1576	BC025692 SH2	n/a	pPRO Ex HTb	His-Grap2 SH2
V1577	BC000631 SH2	n/a	pPRO Ex HTb	His-Grb2 SH2
V1671	BC063035 SH2	V677	V1579	His-loxP-Grap SH2
V1672	BC025692 SH2	V677	V1579	His-loxP-Grap2 SH2
V1673	BC000631 SH2	V677	V1579	His-loxP-Grb2 SH2
V1674	BC006535 SH2	V677	V1579	His-loxP Grb7 SH2

V1675	D86962 SH2	V677	V1579	His-loxP-Grb10 SH2
V1676	BC053559 SH2	V677	V1579	His-loxP-Grb14 SH2
V1800	NM_005902 ORF	V308	V1662	5’-RFP-Smad3
V1854	BC000479 ORF	V308	V1662	5’-RFP-Rab5A
V1856	BC001267 ORF	V308	V1662	5’-RFP-Akt1
